# Comparison of Bone Graft Preparations to Treat a Critical Bone Defect on a Rodent Animal Model

**DOI:** 10.1055/s-0044-1788786

**Published:** 2024-09-04

**Authors:** Rian Souza Vieira, Renan Ernesto Reis Borges, Daniel Guimarães Tiezzi, Antonio Carlos Shimano, Ariane Zamarioli, Helton Luiz Aparecido Defino

**Affiliations:** 1 Departamento de Ortopedia e Anestesiologia, Faculdade de Medicina de Ribeirão Preto, Universidade de São Paulo, Ribeirão Preto, SP, Brasil; 2 Serviço de Cirurgia da Mão, Pontifícia Universidade Católica de Campinas, Campinas, SP, Brasil; 3 Departamento de Ginecologia e Obstetrícia, Divisão de Mastologia e Laboratório de Ciências de Dados Translacionais, Faculdade de Medicina de Ribeirão Preto, Universidade de São Paulo, Ribeirão Preto, SP, Brasil

**Keywords:** bone and bones, bone regeneration, bone transplantation, models, animal

## Abstract

**Objective** Although autologous bone grafting is the most widely used treatment for bone defects, the most effective preparation remains unclear. This animal study aimed to compare different autologous bone grafting preparation for the treatment of rat́s calvaria critical bone defect.

**Methods** 122 rats were randomly allocated into three groups: Simulado, Macerated and Chopped. The specimens underwent craniotomies at the top center of their calvarias with a 7mm diameter circumferential cutter drill. The critical bone defect produced was treated or not according to the group the specimen wasallocated. The rats were euthanized at 3, 6 or 12 weeks post-op and its calvarias were analyzed by histomorphometry, bone densitometry, nanocomputed tomography (nCT), and biomechanical tests.

**Results** The histomorphometry analysis showed the highest percentage of fulfillment of the critical bone defect in the chopped and macerated group when compared to simulado. The densitometry assessment evidenced higher bone mass at all endpoints analysis (p < 0.05) in the chopped group. The nCT data exhibited an expressive increase of bone in the chopped group when compared with the simulado and macerated groups. The biomechanical tests exhibited highest values of deformation, maximum force, and relative stiffness in the chopped group at any time of euthanasia (p < 0.05).

**Conclusions** Our experimental work showed that chopped bone grafting preparation exhibited significant better outcomes than macerated in the treatment of a critical bone defect in rat́s calvaria.

## Introduction

The incidence of bone defects is increasing and demanding solutions. 1 2 3 These injuries have profound economic and clinical impacts, treatment outcomes are limited by high rates of complications. 4 5 6 Autologous bone grafting is the most widely used treatment for bone defects as it is easy to obtain, combines properties and does not induce immune responses nor transmit infections. 7 8 9 However, the most effective autologous bone graft technique is uncertain. 10 11

A critical size bone defect is an orthotopic defect that will not heal without intervention 12 13 or the smallest size of tissue defect that will not completely heal over a lifetime. 12 14 In the rat calvaria model, defects larger than five millimeters are considered critical. 15 16 17

The keystone to the preclinical development of translational technologies is the reliable reproducibility, analogues to the clinical condition they are investigating in animal models. 12 18 We developed an animal based on a rat calvaria critical bone defect treated with different bone graft techniques. Bone regeneration was evaluated in different endpoint periods by histomorphometry, nCT, bone densitometry and biomechanical tests.

This study compared distinct autologous local bone graft treatment techniques used to treat a rat calvaria critical bone defect.

## Materials and Methods

The work was approved by our institution's animal ethics committee under protocol number CEUA: 120/2019

Seven-week-old male Wistar rats weighing 200 g (± 10 g) were obtained from the central animal facility of our institution and housed in individual cages with environmental enrichment in a room with controlled conditions of humidity (55-60%), temperature (23 ± 1°C), and an artificial light/dark cycle of 12 hours. The experimental procedures started after the specimens reached maturity with 300 g (± 10g) and ten-weeks of age.

One hundred and eight (108) rats were randomly divided into three groups ( *n* = 36 per group): (1) Sham: rats with calvaria critical bone defect (7mm in diameter); (2) Macerated: rats with calvaria critical bone defect treated with macerated local autologous bone graft; (3) Chopped: rats with calvaria critical bone defect treated with chopped local autologous bone graft. The experimental procedures (initial surgeries and euthanasia surgeries) were carried out at the same time, surgeon and conditions to minimize biases.

All surgical instruments were sterilized and cooled down to room temperature (23°C). The operating table and instruments were sterilized with ethanol at a 70% concentration.

All rats were anesthetized by intramuscular (IM) injection of Cetamin® by Syntec (Ketamine hydrochloride 10%, 60 mg/kg) and Xylazin® by Syntec (Xylazine hydrochloride 2%, 7.5 mg/kg) and shaved from the bridge of the snout to the distal end of the calvaria. A swab was used to remove hair trimmings and Lacrilube® by Allergan

Inc. applied to each eye. The calvaria was painted with iodine. A sterile surgical field was placed over with a round opening above the calvaria.

A 1.5cm longitudinal incision from superficial skin down to the periosteum over the calvaria, from the nasal bone to caudal to the middle sagittal crest or bregma. The periosteum was divided in half with the scalpel through a sagittal midline elevating it from the skull. A self-retaining retractor spreads the soft tissues and exposes the underlying bone.

**Fig. 1:**
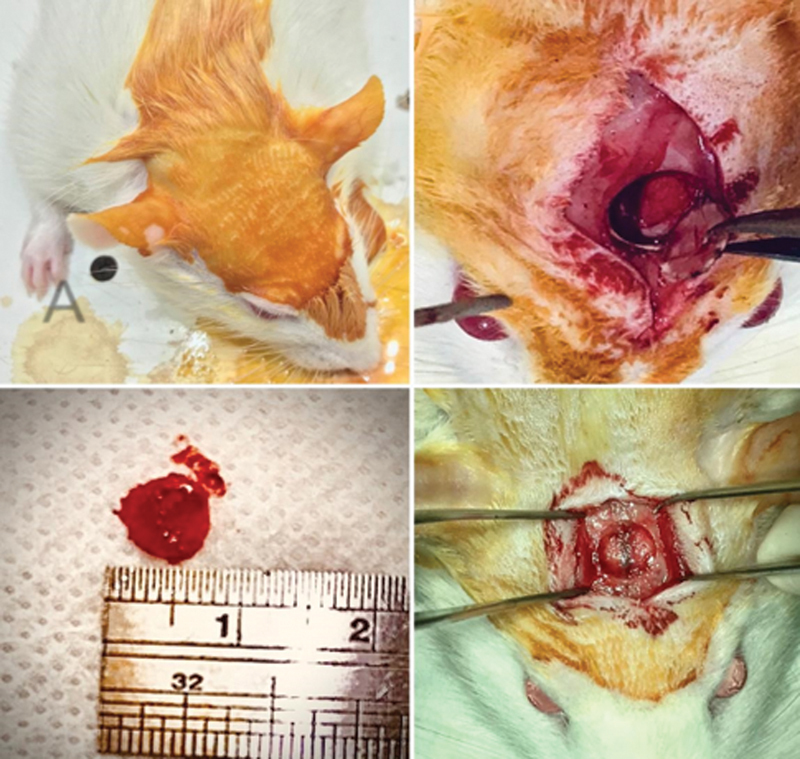
Technical steps of the initial surgery.

A targeted area centered at the intersection of the two calvaria midlines anteroposterior, side to side was drawn. The drill Strong® Micro Motor 210/105L and Zipperer® trephine (7mm total diameter) attached scores the top of the calvaria at 1500 rpm, with sterile saline drops (1 every 2 seconds) to prevent thermal injury. The drill was handled with gentle pressure against the skull surface to produce the defect. An elevator was used into the osteotomy margin completing the defect. The same elevator was used to lift the cylinder of the bone, releasing the dura from the underside of it and pulling the bone out ( Fig. 1 ).

**Fig. 2:**
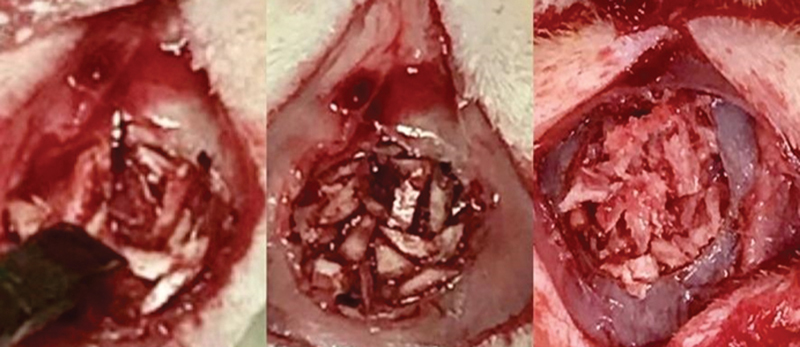
Macerated bone graft preparation filling the critical defect.

**Fig. 3:**
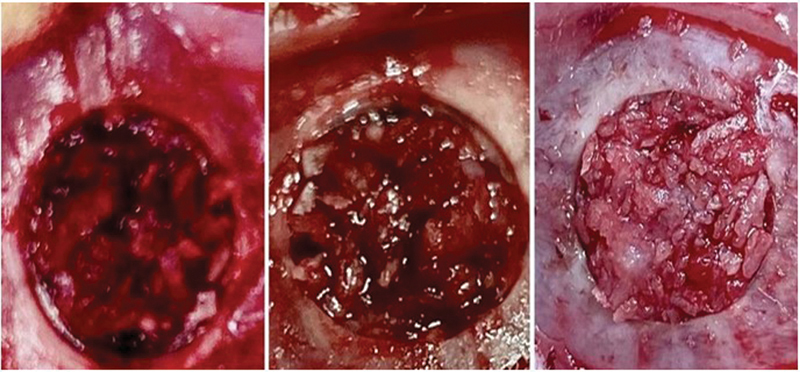
Chopped bone graft preparation filling the critical defect.

The defect was washed with saline to remove debris before grafting ( Figs. 2 and 3 ). The periosteum was closed using a running suture and subsequently the skin using simple gut suture. The rats were placed under observation for any sign of purposeful movement and then transferred to normal husbandry cages.

The daily postoperative care was composed of a health assessment including wound examination, prevention of distress, neurological observation and optimized analgesia. All specimens were treated with tramadol (25 mg/kg) subcutaneously twice daily for 3 days for postoperative pain. The cages were cleaned, and water / food changed three times a week. Rats had their body masses registered routinely once a week. The groups were followed postoperatively and each of them were separated into three subgroups according to the endpoint analysis: 3, 6 and 12 weeks post-surgery.

The rats in all the three groups were sacrificed in three different moments: 3, 6 and 12 weeks post operatively. The euthanasia method used was intramuscular injection of anesthetic overdose of Cetamin® and Xylazin®.

**Fig. 4:**
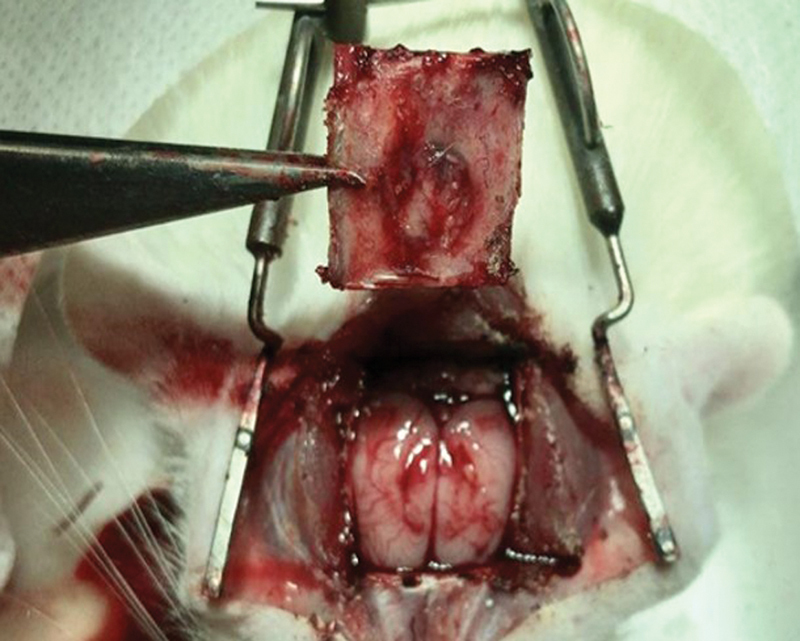
Calvarial rectangle obtained after euthanasia.

Similar initial procedure steps were taken with a slightly longer (2.5cm) surgical access centered in the previous incision. A rectangular segment of the rat calvaria was delimited and cut with a serrated disc SDT® fine granulometry 19/0.15mm at 9000 rpm attached to the same Strong® Micro Motor 210/105L. The calvaria fragments were collected containing the critical bone defect surrounded by original skull bone sized average 15.01 × 11.85 mm ( Fig. 4 ).

A total of 58 samples were designated to histology and were fixed in cold 4% paraformaldehyde while the other 57 samples were embedded in alcohol 70% recipients for bone densitometry assessment, ntomography and mechanical tests.

**Fig. 5:**
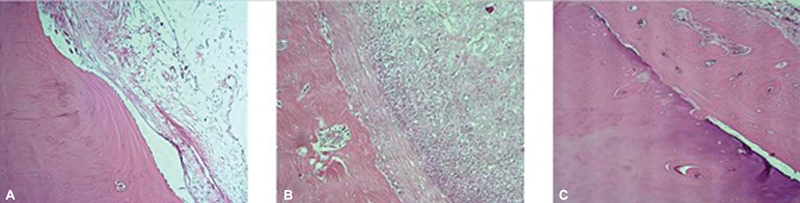
Photomicrographs: 1A sham, 1B Macerated e 1C Chopped (12 weeks post-op).

The routine of decalcification with cold 10% EDTA, progressive dehydration with crescent alcohol concentrations, clearing in xylene with its 3 exchanges and framing in paraffin were performed at the Histology Lab of our Institution. When embedded in paraffin, 5mm sections were obtained and placed on charged histology laminas (Manco Inc., USA). Staining was performed with hematoxylin and eosin (HE) on coronal sections. A bright field microscopy (Axiovert; Carl Zeiss®, Germany) was used to explore those laminas. A CCD camera (AxioCam MRc; Carl Zeiss®, Germany) captured images with many different magnifications for posterior analysis ( Fig. 5 ). A total of 500 histology laminas were produced with these 58 samples.

Bone densitometry analysis was performed at the calvaria samples containing the critical bone defect (total of 57 samples) by Dual-energy X-ray absorptiometry (DXA) using a Lunar® DPX-IQ densitometer (Lunar®; software version 4.7e, GE Healthcare®, United Kingdom). A ROI (region of interest) of approximately 49 mm ^2^ was used to assess the new bone formation. Bone mineral density (BMD, g/cm ^2^ ) and bone mineral content (BMC, g) were measured as protocol.

**Fig. 6:**
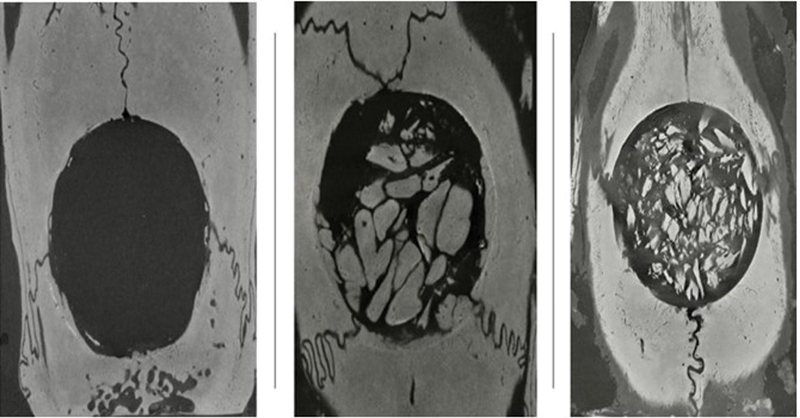
Nanotomographic appearance of the sham, chopped and macerated groups.

The DXA samples were then organized and scanned by the *Nano tomograph Phoenix v|tome|x s – General Electric®* ( Fig. 6 ). Images of each specimen were reconstructed with specific software ( *Dataviewer 1.5.1.2 64bit - SkyScan® Bruker®* ) and analyzed by CTAn ( *CTAn v.1.15.4.0 64bit - SkyScan® Bruker®* ) to determine morphometric parameters in selected regions of interest (ROI). All morphometric parameters are in accordance with the ASBMR nomenclature: Bone Volume (BV), Percent Bone Volume (BV/TV), Bone surface / Volume ratio (BS/BV), Structure model index (SMI), Trabecular Thickness (Tb.Th), Trabecular number (Tb.N), Trabecular separation (Tb.Sp), Total porosity percent (Po tot) and Connectivity density (Conn.Dn).

**Fig. 7:**
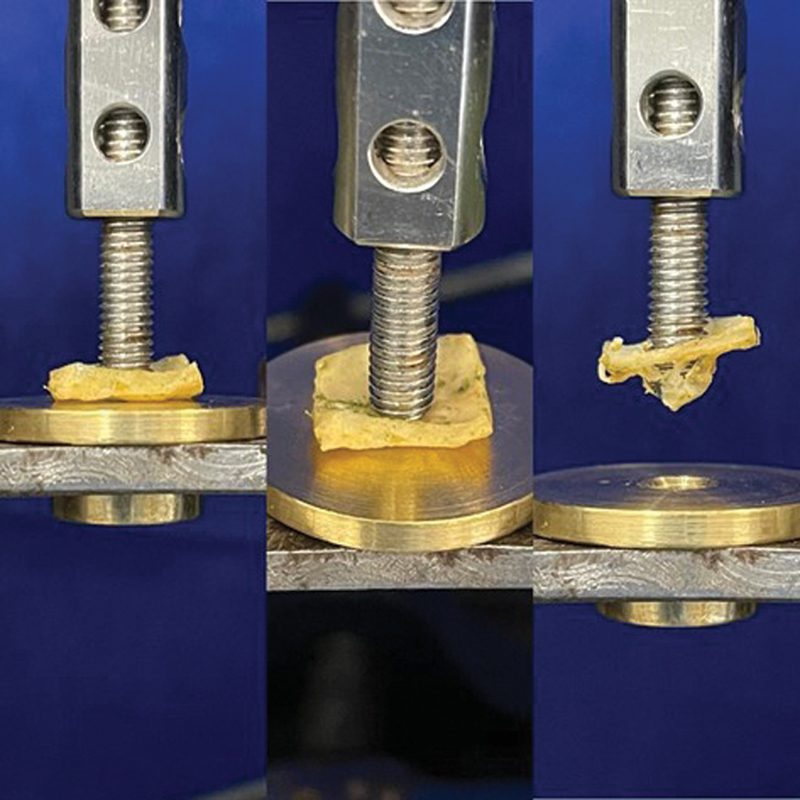
Mechanical testing from different angles and in detail.

The 57 calvaria samples were mechanically stressed in a perforation pushout test. A universal testing machine was used for critical bone defect stress tests with a 50N load cell *(Trd 28–EMIC DL 10000®)* . The settings of the tests were: 1mm/min progression speed, 1N pre-load and 30s accommodation time. The bone sample was positioned on a custom-made metallic support with an 8mm diameter circumferential hole centered and aligned with the critical bone defect center. A cylindrical metal pusher with 7mm diameter also centered and aligned with the bone defect and the support circumferential hole center gradually descended to contact the samples, preload and then gradually stress the with or without graft until complete disruption ( Fig. 7 ). Using the Tesc 3.04 software script, raw data were filtered and measured maximum force, deformation, relative stiffness and elasticity maximum strength. The samples were hydrated with saline during the tests.

Hypothesis tests were performed to analyze the variation on bone regeneration across different bone engraftment techniques compared to the sham group. All variables were tested for normality based on visual histogram and the Shapiro-Wilks test. Descriptive statistics used the median and median absolute deviation (MAD) as measures of central tendency and dispersion, respectively. We applied the nonparametric KruskalWallis test followed by the post hoc Dunn for Kruskal-Wallis multiple comparison. The Spearman rank correlation index was calculated to analyze the relationship between multiple parameters obtained by nCT, bone densitometry and the stress test. All statistical analyses were performed in R for Linux/GNU version 4.1.0 and the p value < 0.05 was considered statistically significant.

## Results

A qualitative histological analysis evidenced a higher proportion of new bone formation in the critical bone defect of the grafted groups when compared to the shams. Furthermore, the chopped group is suggested to induce the uppermost new bone formation of all under bright field microscopy qualitative analysis.

In accordance, our quantitative analysis proved a higher percentage of new bone formed in the Chopped and Macerated groups when compared to Sham (p = 0.008). Although the Chopped group showed the highest percentages of new bone formation, the difference with the Macerate group was not statistically relevant (p = 0.1) ( Fig. 4 ). We understand this observed trend deserves attention.

**Fig. 8:**
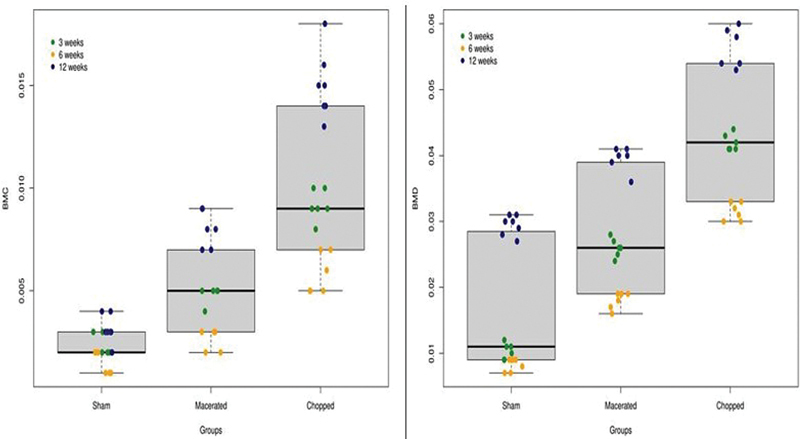
Bone mineral concentration (BMC) and bone mineral density (DMO) in groups and times.

We observed a significant overall increase in BMD by comparing the different endpoint periods (p < 0.001) ( Table 1 ). At 3 weeks after the procedure, the median BMD was 0.01 in the sham group (MAD = 0.001), 0.026 (MAD = 0.001) in the macerated group, and 0.041 (MAD = 0.0007) in the chopped group (p = 0.0004). The differences persisted at 6 and 12 weeks (p = 0.0004 and p = 0.0002, respectively). In the sham group the median BMD was 0.0085 (MAD = 0.0007) and 0.03 (MAD = 0.001) at 6 and 12 weeks, respectively. In the macerated group the median BMD was 0.0185 (MAD = 0.0007) and 0.04 (MAD = 0.001), and in the chopped group the median BMD was 0.031 (MAD = 0.002) and 0.054 (MAD = 0.001) at 6 and 12 weeks, respectively ( Fig. 8 ). The post hoc test for pairwise comparison has demonstrated the increment in bone regeneration comparing the chopped group with the sham.

**Table 1:** Densitometry statistics summary

Variable	Weeks	Groups	Median	MAD	P Value
BMC	3w				0.00038150651248
		Sham	0.002	0	
		Chopped	0.009	0.0007413	
		Macerated	0.005	0	
BMC	6w				0.00067991117923
		Sham	0.0015	0.0007413	
		Chopped	0.0055	0.0007413	
		Macerated	0.003	0	
BMC	12w				0.00019298618864
		Sham	0.003	0	
		Chopped	0.015	0.0014826	
		Macerated	0.008	0.0014826	
BMD	3w				0.00048367583690
		Sham	0.0105	0.0014826	
		Chopped	0.0415	0.0007413	
		Macerated	0.026	0.0014826	
BMD	6w				0.00046850172588
		Sham	0.0085	0.0007413	
		Chopped	0.0315	0.0022239	
		Macerated	0.0185	0.0007413	
BMD	12w				0.00020323763567
		Sham	0.03	0.0014826	
		Chopped	0.054	0.0014826	
		Macerated	0.04	0.0014826	

BMC, Bone mass content; BMD, Bone mineral content.

**Fig. 9:**
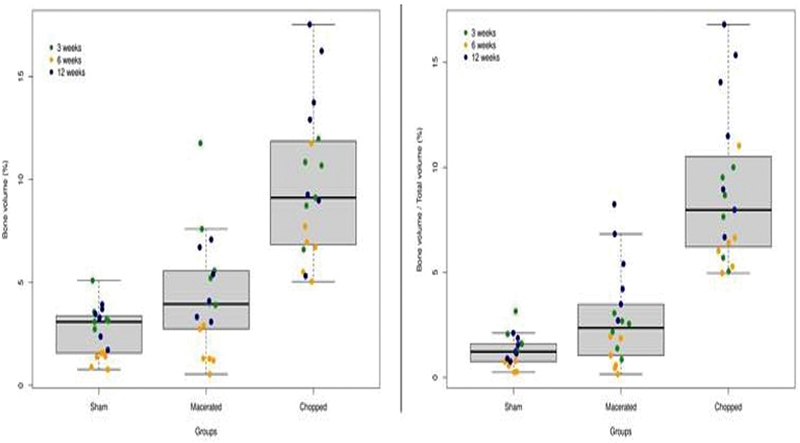
Bone Volume (BV) and Bone Volume by Total Volume (BV/TV).

We analyzed the nCT parameters across the experimental groups. The BV and BV/TV estimate the volume of bone regenerated in the critical bone defect area. We observed a significant overall bone regeneration in chopped and macerated groups compared to the sham group (p < 0.0001) ( Fig. 9 ). The median BV in the sham group at 3, 6 and 12 weeks were 1.4 (MAD= 0.2), 3.1 (MAD= 0.35) and 3.3 (MAD= 0.6), respectively. In the macerated group the median BV at 3, 6 and 12 weeks were 1.3 (MAD= 0.6), 5.4 (MAD= 2.1) and 4.7 (MAD= 2.3) ( Table 2 ).

**Table 2:** Nano tomography statistics summary

Variable	Weeks	Group	Median	P Value
BV	3w			0,002338
		Sham	3,166115	
		Chopped	9,8977	
		Macerated	5,39414	
BV	6w			0,0033992
		Sham	1,39563	
		Chopped	6,83119	
		Macerated	1,30313	
BV	12w			0,0013393
		Sham	3,29243	
		Chopped	12,90203	
		Macerated	4,74866	
BV/TV	3w			0,0030956
		Sham	1,60475	
		Chopped	8,1638	
		Macerated	2,360649	
BV/TV	6w			0,0029369
		Sham	0,622355	
		Chopped	6,21231	
		Macerated	0,812585	
BV/TV	12w			0,0003588
		Sham	1,22118	
		Chopped	11,49151	
		Macerated	4,80795	

BV, Bone Volume; TV, Toal Volume.

We analyzed the correlation between the BMD and microstructural parameters obtained by nCT and detected a high positive correlation with BV, BV/TV and Tb.N (rho= 0.77, rho= 0.82 and rh0= 0.81, respectively), and a strong anticorrelation with trabecular separation and total porosity (rho= -0.73 and rho= -0.82, respectively).

**Fig. 10:**
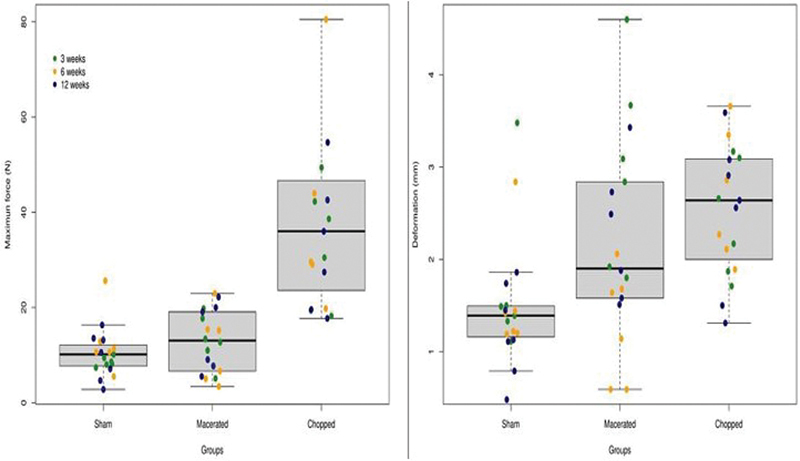
Maximum force (N) and deformation (mm).

The biomechanical analysis was performed to infer the behavior of the new bone formation under a stress test. We measured the maximum force, the deformation, the relative stiffness and the elasticity maximum strength for every calvaria specimen. We observed that the calvaria specimens from rats subjected to the engraftment with chopped bone were significantly more resistant to the stress ( Table 3 ) (p < 0.0001 for all variables). The overall median (MAD) maximum force, the deformation, the relative stiffness and the elasticity maximum strength in the chopped group was 36 (19.9), 2.6 (0.8), 12.5 (5.5) and 39.1 (20.5), respectively, compared to 10.1 (4), 1.39 (0.3), 6.8 (3) and 6.8 (2.8) in sham and 13.1 (9.2), 1.9 (1), 4.7 (3) and 14.6 (11) in macerated groups ( Fig. 10 ).

**Table 3:** Biomechanical test statistics summary

Variable	Weeks	Grupo	Median	P Value
Maximum strenght	3w			0,002338
		Sham	8,375	
		Chopped	34,54	
		Macerated	13,06	
Maximum strenght	6w			0,006104
		Sham	11	
		Chopped	36,77	
		Macerated	10,92	
Maximum strenght	12w			0,003498
		Sham	10,56	
		Chopped	35,99	
		Macerated	14,075	
Deformation	3w			0,041776
		Sham	1,44	
		Chopped	2,415	
		Macerated	2,965	
Deformation	6w			0,010158
		Sham	1,32	
		Chopped	2,565	
		Macerated	1,39	
Deformation	12w			0,014718
		Sham	1,13	
		Chopped	2,64	
		Macerated	2,185	

## Discussion

Bone defects represent relevant challenges to Orthopedists. 1 2 3 4 19 20 The treatments for critical bone defects often result in complications. 4 5 6 There is also an association with prolonged recovery decreasing quality of life. 21

The success of bone grafting is limited by many factors such as remodeling capacities of the host, material itself, surgical technique 22 and even how the graft is handled. 23 24 Autografting is the most widely used treatment for bone defects, but it remains unclear what preparation technique will induce the greater regeneration. 8 24 25 It is well stablished that graft dimensions influence the outcome of treatment 26 27 28 and the calvaria has already been described as a donor site 29 but, to the best of our knowledge, there was not a study comparing macerated and chopped local autologous bone graft preparations.

From macroscopic and clinical to microstructural level, our data demonstrated the higher efficiency of chopped bone graft preparation at inducing bone regeneration.

Macroscopically, we detected higher bone mass (density and content) in the Chopped group when compared to the Sham and Macerated at all endpoints: three, six and twelve-weeks following surgery. Likewise, our mechanical tests also confirmed the higher effectiveness of chopped bone graft at inducing bone regeneration with stronger properties. As of note, the chopped group exhibited newly formed bone with higher maximum force, stiffness, and deformation, which demands heavier loads prior to failure and has a higher modulus of elasticity. Histologically, our qualitative analysis suggests a much higher percentage of woven bone formation and critical bone defect fulfillment in the Chopped samples than in the Macerated group, which could be confirmed microscopically by our nCT assessment. The Chopped group exhibited a remarkable osteogenic effect at increasing the bone formation-related parameters of bone volume and fraction, connectivity density, trabecular thickness and number, concomitant with decreases in resorption-related parameter; bone surface/volume ratio, structure model index, trabecular separation and porosity.

It is important to highlight that models of rat calvaria critical bone defect can be used for the study of bone regeneration and biomaterials before considering larger animals or future potential human applications. 30 The first rat calvaria model of a bone defect was described in 1973 by Freeman and Turnbull but only accomplished by Takagi and Urist in 1982. We confirmed the efficacy of our model as no spontaneous bone regeneration occurred in the Sham group at any endpoint.

Among the limitations of our study, we may include a lack in elucidating the mechanisms leading to a better bone regeneration due to chopped bone graft preparation. Although it was not the purpose of our study, future studies should include molecular assessment to expand our critical capacity and data scientific production, as well as to target potent pharmacological therapies to further induce regeneration. The comparison among local autologous bone graft, distant autologous bone graft, bone substitutes adjuvants should be further investigated in the bone healing processes to reach the best available critical bone defect treatment.

## Conclusion

This study evidenced that different techniques to prepare and locally treat bone defects may play an important role at inducing bone regeneration. We used an animal model of bone defect that did not spontaneously heal throughout the experiment. Conversely, our protocols for local autologous bone graft induced bone regeneration in this animal model. The chopped bone graft exhibited newly formed bone with higher mass, improved microarchitecture and better mechanical integrity than the newly formed bone followed by macerated bone graft.
