# Supplementary material for: Comparison of Bone Graft Preparations to Treat a Critical Bone Defect on a Rodent Animal Model
Source: Rev Bras Ortop (Sao Paulo). 2024 Sep 4;59(4):e532–41. doi: 10.1055/s-0044-1788786 (PMC11374410; doi:10.1055/s-0044-1788786)
Supplement: Related texts

# Research article: Comparação de preparações de enxertos ósseos para tratamento de defeitos ósseos críticos em um modelo animal roedor

**Keywords:** modelos animais, osso e ossos, regeneração óssea, transplante ósseo

## Abstract

**Objetivo** Embora enxertos ósseos autólogos sejam o tratamento mais utilizado para defeitos ósseos, a preparação mais eficaz ainda é obscura. Este estudo animal teve como objetivo comparar diferentes preparações de enxerto ósseo autólogo para o tratamento de defeito ósseo crítico no crânio de ratos.

**Métodos** No total, 122 ratos foram alocados aleatoriamente em três grupos: Simulado, enxerto macerado e enxerto picado. Os espécimes foram submetidos a craniotomias no centro superior do crânio com broca de corte circunferencial de 7 mm de diâmetro. O defeito ósseo crítico produzido foi tratado ou não de acordo com o grupo de alocação do animal. Os ratos foram eutanasiados às 3, 6 ou 12 semanas após a cirurgia e seus crânios foram analisados por histomorfometria, densitometria óssea, nanotomografia computadorizada (nTC) e testes biomecânicos.

**Resultados** A análise histomorfométrica mostrou maior percentual de preenchimento do defeito ósseo crítico no grupo picado e macerado em comparação ao simulado. A avaliação densitométrica evidenciou maior massa óssea em todos os desfechos de análise (p < 0,05) no grupo picado. Os dados de nTC revelaram um aumento ósseo expressivo no grupo picado em comparação aos grupos simulado e macerado. Os testes biomecânicos mostraram maiores valores de deformação, resistência máxima e rigidez relativa no grupo picado em qualquer momento da eutanásia (p < 0,05).

**Conclusões** Nosso estudo mostrou que a preparação de enxerto ósseo picado gerou resultados significativamente melhores do que os enxertos macerados no tratamento de defeitos ósseos críticos no crânio de ratos.

## Introdução

A incidência de defeitos ósseos tem aumentado e exige soluções. 1 2 3 Essas lesões têm profundos impactos econômicos e clínicos; além disso, os desfechos do tratamento são limitados pelas altas taxas de complicações. 4 5 6 O enxerto ósseo autólogo é o tratamento mais utilizado para defeitos ósseos por ser fácil de obter, combinar propriedades e não induzir respostas imunes ou transmitir infecções. 7 8 9 No entanto, não se sabe a técnica de enxerto ósseo autólogo mais eficaz. 10 11

Um defeito ósseo de tamanho crítico é um defeito ortotópico que não cicatriza sem intervenção 12 13 ou o defeito tecidual de menor tamanho que não cicatriza por completo ao longo da vida. 12 14 No modelo de crânio de rato, defeitos maiores que 5 mm são considerados críticos. 15 16 17

A pedra angular do desenvolvimento pré-clínico de tecnologias translacionais é a reprodutibilidade confiável, análoga à condição clínica investigada em modelos animais. 12 18 Desenvolvemos um modelo animal de defeito ósseo crítico em crânios de ratos e seu tratamento com diferentes técnicas de enxerto ósseo. A regeneração óssea foi avaliada em diferentes tempos por histomorfometria, nanotomografia computadorizada (nTC), densitometria óssea e testes biomecânicos.

Este estudo comparou diferentes técnicas de tratamento de defeitos ósseos críticos no crânio de ratos com enxertos ósseos autólogos locais.

## Materiais e Métodos

Este estudo foi aprovado pelo comitê de ética animal de nossa instituição sob número de protocolo CEUA 120/2019.

Ratos Wistar machos com 7 semanas de idade, pesando 200 g (± 10 g) foram obtidos do biotério central de nossa instituição e alojados em gaiolas individuais com enriquecimento ambiental em sala com condições controladas de umidade (55 a 60%), temperatura (23 ± 1 C) e ciclo claro/escuro artificial de 12 horas. Os procedimentos experimentais começaram depois que os espécimes atingiram a maturidade, com 300 g (±10 g) e 10 semanas de idade.

Cento e oito (108) ratos foram divididos aleatoriamente em três grupos (36 animais por grupo): (1) grupo simulado: ratos com defeito ósseo crítico no crânio (7 mm de diâmetro); (2) grupo macerado: ratos com defeito ósseo crítico no crânio tratados com enxerto ósseo autólogo local macerado; (3) grupo picado: ratos com defeito ósseo crítico no crânio tratados com enxerto ósseo autólogo local picado. Os procedimentos experimentais (primeiras cirurgias e cirurgias de eutanásia) foram realizados ao mesmo tempo, pelo mesmo cirurgião e nas mesmas condições para minimizar vieses.

Todos os instrumentos cirúrgicos foram esterilizados e resfriados à temperatura ambiente (23 C). A mesa cirúrgica e os instrumentos foram esterilizados com etanol a 70%.

Todos os ratos foram anestesiados por injeção intramuscular (IM) de Cetamin® da Syntec (cloridrato de cetamina 10%, 60 mg/kg) e Xylazin® da Syntec (cloridrato de xilazina 2%, 7,5 mg/kg) e tricotomizados da ponte nasal até a extremidade distal do crânio. Um cotonete foi utilizado para retirada de aparas de pelos e Lacrilube® da Allergan Inc. foi aplicado em cada olho. O crânio foi pintado com iodo. Um campo cirúrgico estéril foi colocado com uma abertura redonda acima do crânio.

Uma incisão longitudinal de 1,5 cm da pele superficial foi estendida até o periósteo sobre o crânio, do osso nasal até caudal à crista sagital média ou bregma. O periósteo foi dividido ao meio com o bisturi por meio de uma linha média sagital, elevando-o do crânio. Um afastador automático retraiu os tecidos moles e expos o osso subjacente.

Uma área alvo centralizada na intersecção das duas linhas médias do crânio anteroposterior, de lado a lado, foi desenhada. Uma broca Strong® Micro Motor 210/105L e uma trefina Zipperer® (7 mm de diâmetro total) fixadas marcaram o topo do crânio a 1.500 rpm, com aplicação de salina estéril em gotas (uma gota a cada 2 segundos) para evitar lesão térmica. A broca foi manuseada com leve pressão contra a superfície do crânio para produzir o defeito. Um elevador foi utilizado na margem da osteotomia completando o defeito. O mesmo elevador foi usado para levantar o cilindro do osso, liberando a dura-máter da parte inferior e puxando o osso para fora ( Fig. 1 ).

**Fig. 1:** Etapas técnicas da primeira cirurgia.

O defeito foi lavado com solução salina para remoção de detritos antes do enxerto ( Figs. 2 e 3 ). O periósteo foi fechado com sutura contínua e a pele recebeu sutura simples com categute. Os ratos foram colocados sob observação quanto a quaisquer sinais de movimento proposital e depois transferidos para as gaiolas normais de criação.

**Fig. 2:** Preparação de enxerto ósseo macerado para preenchimento do defeito crítico.

**Fig. 3:** Preparação de enxerto ósseo picado para preenchimento do defeito crítico.

Os cuidados pós-operatórios diários foram compostos por avaliação de saúde, incluindo exame de feridas, prevenção de sofrimento, observação neurológica e analgesia otimizada. Todos os animais foram tratados com tramadol (25 mg/kg) por via subcutânea duas vezes ao dia durante 3 dias para evitar dor pós-operatória. As gaiolas foram limpas e a água/comida trocada três vezes por semana. Os ratos tiveram suas massas corporais registradas uma vez por semana. Os grupos foram acompanhados no pós-operatório e cada um deles foi separado em três subgrupos de acordo com a análise do desfecho, às 3, 6 e 12 semanas após a cirurgia.

Os ratos dos três grupos foram eutanasiados em três momentos diferentes, às 3, 6 e 12 semanas após as cirurgias. O método de eutanásia utilizado foi a injeção intramuscular de *overdose* anestésica de Cetamin® e Xylazin®.

Procedimentos iniciais semelhantes foram realizados com acesso cirúrgico um pouco maior (2,5 cm) centrado na incisão anterior. Um segmento retangular do crânio do rato foi delimitado e seccionado com disco serrilhado SDT® de granulometria fina 19/0,15 mm a 9.000 rpm acoplado ao mesmo Strong® Micro Motor 210/105L. Os fragmentos de crânio contendo o defeito ósseo crítico circundado por osso craniano original, com tamanho médio de 15,01 × 11,85 mm, foram coletados ( Fig. 4 ).

**Fig. 4:** Retângulo do crânio obtido após a eutanásia.

Um total de 58 amostras foram destinadas à histologia e fixadas com paraformaldeído a 4% frio, enquanto as outras 57 amostras foram fixadas com álcool a 70% para avaliação de densitometria óssea, nTC e testes mecânicos.

A rotina de descalcificação com ácido etilenodiamino tetra-acético (EDTA) a 10% frio, desidratação progressiva com concentrações crescentes de álcool, clarificação em xilol em três trocas e emblocamento em parafina foi realizada no Laboratório de Histologia de nossa Instituição. Após a inclusão em parafina, cortes de 5 mm foram obtidos e colocados em lâminas histológicas carregadas (Manco Inc., EUA). A coloração dos cortes coronais utilizou hematoxilina e eosina (HE). Um microscópio de campo claro (Axiovert; Carl Zeiss®, Alemanha) foi utilizado para análise das lâminas. Uma câmera CCD (AxioCam MRc; Carl Zeiss®, Alemanha) capturou imagens com diversas ampliações para interpretação posterior ( Fig. 5 ). No total, 500 lâminas histológicas foram produzidas com essas 58 amostras.

**Fig. 5:** Fotomicrografias: 1A, grupo simulado; 1B, grupo macerado; 1C, grupo picado (12 semanas após a cirurgia).

A análise de densitometria óssea foi realizada nas 57 amostras de crânio contendo o defeito ósseo crítico por absorciometria de raios X de dupla energia (DXA) usando um densitômetro Lunar® DPX-IQ (Lunar®; *software* versão 4.7e, GE Healthcare®, Reino Unido). Uma região de interesse (ROI) de aproximadamente 49 mm ^2^ foi utilizada para avaliação da nova formação óssea. A densidade mineral óssea (BMD, g/cm ^2^ ) e o conteúdo mineral ósseo (BMC, g) foram medidos conforme protocolo.

As amostras de DXA foram então organizadas e escaneadas com *Nano tomograph Phoenix v|tome|x s –* General Electric *®* ( Fig. 6 ). As imagens de cada espécime foram reconstruídas com *software* específico (Dataviewer 1.5.1.2 64 bits - SkyScan® Bruker®) e analisadas por CTAn (CTAn v.1.15.4.0 64 bits - SkyScan® Bruker®) para determinar parâmetros morfométricos nas ROIs selecionadas. Todos os parâmetros morfométricos estão de acordo com a nomenclatura da *American Society for Bone and Mineral Research* (ASBMR): volume ósseo (BV), volume ósseo percentual (BV/TV), relação superfície/volume ósseo (BS/BV), índice de modelo de estrutura (SMI), espessura trabecular (Tb.Th), número trabecular (Tb.N), separação trabecular (Tb.Sp), porcentagem de porosidade total (Po tot) e densidade de conectividade (Conn.Dn).

**Fig. 6:** Aparência nanotomográfica dos grupos simulado, picado e macerado.

As 57 amostras de crânio foram tensionadas mecanicamente em um teste de perfuração do tipo *push-out* . Os testes de estresse de defeitos ósseos críticos ocorreram em equipamento universal com célula de carga de 50 N (Trd 28–EMIC DL 10000®). As configurações dos testes foram velocidade de progressão de 1 mm/min, pré-carga de 1 N e tempo de acomodação de 30 s. A amostra óssea foi posicionada sobre um suporte metálico customizado com furo circunferencial de 8 mm de diâmetro centralizado e alinhado com o centro do defeito ósseo crítico. Um empurrador metálico cilíndrico com 7 mm de diâmetro também centrado e alinhado com o defeito ósseo e o centro do furo circunferencial de suporte desceu gradativamente até entrar em contato com as amostras, pré-carregar e então tensionar gradativamente as amostras com ou sem enxerto até o rompimento completo ( Fig. 7 ). Utilizando o *script* do *software* Tesc 3.04, os dados brutos foram filtrados e medidas de força máxima, deformação, rigidez relativa e resistência máxima de elasticidade. As amostras foram hidratadas com solução salina durante os testes.

**Fig. 7:** Testes mecânicos de diferentes ângulos e em detalhes.

Os testes de hipóteses analisaram a variação na regeneração óssea com as diferentes técnicas de enxerto ósseo em comparação com o grupo simulado. Todas as variáveis foram testadas quanto à normalidade com base no histograma visual e no teste de Shapiro-Wilks. A estatística descritiva utilizou a mediana e o desvio absoluto da mediana (MAD) como medidas de tendência central e dispersão, respectivamente. Aplicou-se o teste não paramétrico de Kruskal-Wallis seguido do teste post-hoc de Dunn para comparação múltipla de Kruskal-Wallis. O índice de correlação de Spearman foi calculado para analisar a relação entre os múltiplos parâmetros obtidos por nTC, densitometria óssea e testes de estresse. Todas as análises estatísticas foram realizadas em R para Linux/GNU versão 4.1.0 e o valor de p < 0,05 foi considerado estatisticamente significativo.

## Resultados

Uma análise histológica qualitativa evidenciou maior proporção de neoformação óssea no defeito ósseo crítico dos grupos enxertados em comparação ao grupo simulado. Além disso, a análise qualitativa de microscopia de campo claro sugeriu que o grupo picado induzia a maior formação óssea de todas.

Da mesma forma, a análise quantitativa comprovou o maior percentual de osso novo formado nos grupos picado e macerado em comparação ao grupo simulado (p = 0,008). Embora o grupo picado tenha apresentado os maiores percentuais de neoformação óssea, a diferença com o grupo macerado não foi estatisticamente relevante (p = 0,1) ( Fig. 4 ). Acreditamos que essa tendência mereça atenção.

Observamos um aumento geral significativo na BMD ao comparar os diferentes períodos de desfecho (p < 0,001) ( Tabela 1 ). Três semanas após o procedimento, a mediana da BMD foi de 0,01 (MAD = 0,001) no grupo simulado, 0,026 (MAD = 0,001) no grupo macerado e 0,041 (MAD = 0,0007) no grupo picado (p = 0,0004). As diferenças persistiram às 6 e 12 semanas (p = 0,0004 e p = 0,0002, respectivamente). No grupo simulado, a BMD mediana foi de 0,0085 (MAD = 0,0007) e 0,03 (MAD = 0,001) às 6 e 12 semanas, respectivamente. No grupo macerado, a BMD mediana foi 0,0185 (MAD = 0,0007) e 0,04 (MAD = 0,001) e, no grupo picado, a BMD mediana foi 0,031 (MAD = 0,002) e 0,054 (MAD = 0,001) às 6 e 12 semanas, respectivamente ( Fig. 8 ). O teste post-hoc para comparação pareada demonstrou o incremento na regeneração óssea no grupo picado em comparação ao grupo simulado.

**Fig. 8:** Concentração mineral óssea (BMC) e densidade mineral óssea (BMD) por grupos e tempos.

**Tabela 1:** Resumo estatístico de densitometria

Variável	Semanas	Grupos	Mediana	MAD	Valor de p
BMC	3				0.00038150651248
		Simulado	0,002	0	
		Picado	0,009	0,0007413	
		Macerado	0,005	0	
BMC	6				0,00067991117923
		Simulado	0,0015	0,0007413	
		Picado	0,0055	0,0007413	
		Macerado	0,003	0	
BMC	12				0,00019298618864
		Simulado	0,003	0	
		Picado	0,015	0,0014826	
		Macerado	0,008	0,0014826	
BMD	3				0,00048367583690
		Simulado	0,0105	0,0014826	
		Picado	0,0415	0,0007413	
		Macerado	0,026	0,0014826	
BMD	6				0,00046850172588
		Simulado	0,0085	0,0007413	
		Picado	0,0315	0,0022239	
		Macerado	0,0185	0,0007413	
BMD	12				0,00020323763567
		Simulado	0,03	0,0014826	
		Picado	0,054	0,0014826	
		Macerado	0,04	0,0014826	

BMC, Conteúdo de massa óssea; BMD, conteúdo mineral ósseo, MAD, desvio absoluto da mediana.

Analisamos os parâmetros nTC nos grupos experimentais. O BV e o BV/TV estimam o volume de osso regenerado na área do defeito ósseo crítico. Observamos uma regeneração óssea global significativa nos grupos picado e macerado em comparação ao grupo simulado (p < 0.0001) ( Fig. 9 ). O BV mediano do grupo simulado às 3, 6 e 12 semanas foi de 1,4 (MAD = 0,2), 3,1 (MAD = 0,35) e 3,3 (MAD = 0,6), respectivamente. No grupo macerado, o BV mediano às 3, 6 e 12 semanas foi de 1,3 (MAD = 0,6), 5,4 (MAD = 2,1) e 4,7 (MAD = 2,3) ( Tabela 2 ).

**Tabela 2:** Resumo estatístico de nanotomografia

Variável	Semanas	Grupo	Mediana	Valor de p
BV	3			0,002338
		Simulado	3,166115	
		Picado	9,8977	
		Macerado	5,39414	
BV	6			0,0033992
		Simulado	1,39563	
		Picado	6,83119	
		Macerado	1,30313	
BV	12			0,0013393
		Simulado	3,29243	
		Picado	12,90203	
		Macerado	4,74866	
BV/TV	3			0,0030956
		Simulado	1,60475	
		Picado	8,1638	
		Macerado	2,360649	
BV/TV	6			0,0029369
		Simulado	0,622355	
		Picado	6,21231	
		Macerado	0,812585	
BV/TV	12			0,0003588
		Simulado	1,22118	
		Picado	11,49151	
		Macerado	4,80795	

BV, Volume ósseo; TV, volume total.

**Fig. 9:** Volume ósseo (BV) e volume ósseo por volume total (BV/TV).

Analisamos a correlação entre a BMD e os parâmetros microestruturais obtidos por nTC e detectamos uma alta correlação positiva com BV, BV/TV e Tb.N (rho = 0,77, rho = 0,82 e rho = 0,81, respectivamente), e uma forte anticorrelação com a separação trabecular e a porosidade total (rho = -0,73 e rho = -0,82, respectivamente).

A análise biomecânica inferiu o comportamento da neoformação óssea sob testes de estresse. Medimos a força máxima, a deformação, a rigidez relativa e a resistência máxima à elasticidade de cada amostra de crânio. Observamos que os espécimes de crânio de ratos submetidos ao enxerto com osso picado foram significativamente mais resistentes ao estresse ( Tabela 3 ) (p < 0,0001 para todas as variáveis). A força máxima mediana geral (MAD), a deformação, a rigidez relativa e a resistência máxima à elasticidade no grupo picado foram de 36 (19,9), 2,6 (0,8), 12,5 (5,5) e 39,1 (20,5), respectivamente, em comparação a 10,1 (4), 1,39 (0,3), 6,8 (3) e 6,8 (2,8) no grupo simulado e 13,1 (9,2), 1,9 (1), 4,7 (3) e 14,6 (11) no grupo macerado ( Fig. 10 ).

**Fig. 10:** Resistência máxima (N) e deformação (mm).

**Tabela 3:** Resumo estatístico de testes biomecânicos

Variável	Semanas	Grupo	Mediana	Valor de p
Resistência máxima	3			0,002338
		Simulado	8,375	
		Picado	34,54	
		Macerado	13,06	
Resistência máxima	6			0,006104
		Simulado	11	
		Picado	36,77	
		Macerado	10,92	
Resistência máxima	12			0,003498
		Simulado	10,56	
		Picado	35,99	
		Macerado	14,075	
Deformação	3			0,041776
		Simulado	1,44	
		Picado	2,415	
		Macerado	2,965	
Deformação	6			0,010158
		Simulado	1,32	
		Picado	2,565	
		Macerado	1,39	
Deformação	12			0,014718
		Simulado	1,13	
		Picado	2,64	
		Macerado	2,185	

## Discussão

Os defeitos ósseos representam desafios relevantes para os ortopedistas. 1 2 3 4 19 20 Os tratamentos de defeitos ósseos críticos muitas vezes geram complicações. 4 5 6 Há também uma associação com recuperação prolongada e diminuição da qualidade de vida. 21

O sucesso do enxerto ósseo é limitado por muitos fatores, como a capacidade de remodelação do hospedeiro, o próprio material, a técnica cirúrgica 22 e até mesmo a forma de manuseio do enxerto. 23 24 O autoenxerto é o tratamento mais utilizado para defeitos ósseos, mas ainda não está claro qual técnica de preparo induz maior regeneração. 8 24 25 Está bem estabelecido que as dimensões do enxerto influenciam o resultado do tratamento 26 27 28 e o crânio já foi descrito como a área doadora, 29 mas, até onde sabemos, não houve um estudo comparando preparações de enxerto ósseo autólogo local macerado e picado.

Do ponto de vista macroscópico e clínico ao microestrutural, nossos dados demonstraram a maior eficiência da preparação do enxerto ósseo picado na indução da regeneração óssea.

Macroscopicamente, detectamos maior massa óssea (densidade e conteúdo) no grupo picado em comparação aos grupos simulado e macerado em todos os desfechos (3, 6 e 12 semanas após a cirurgia). Da mesma forma, nossos testes mecânicos também confirmaram a maior eficácia do enxerto ósseo picado na indução da regeneração óssea com propriedades mais fortes. Vale ressaltar que o grupo picado apresentou ossos recém-formados com maior força máxima, rigidez e deformação, exigindo cargas maiores antes da falha e demonstrando maior módulo de elasticidade. Histologicamente, nossa análise qualitativa sugere uma porcentagem muito maior de formação óssea tecida e preenchimento de defeitos ósseos críticos nas amostras do grupo picadas do que no grupo macerado, o que pode ser confirmado microscopicamente pela avaliação por nTC. O grupo picado exibiu um efeito osteogênico notável no aumento dos parâmetros relacionados à formação de volume ósseo e fração óssea, densidade de conectividade, espessura e número trabecular, além de diminuições nos parâmetros relacionados à reabsorção, como relação superfície/volume ósseo, índice de modelo de estrutura, separação trabecular e porosidade.

É importante destacar que modelos de defeito ósseo crítico no crânio de ratos podem ser usados para o estudo de regeneração óssea e biomateriais antes de considerar animais maiores ou futuras aplicações humanas. 30 O primeiro modelo de defeito ósseo no crânio de ratos foi descrito em 1973 por Freeman e Turnbull, mas realizado por Takagi e Urist somente em 1982. Confirmamos a eficácia do nosso modelo, pois não houve regeneração óssea espontânea no grupo simulado em qualquer desfecho.

Dentre as limitações do nosso estudo, podemos incluir a falta de elucidação dos mecanismos que levam a uma melhor regeneração óssea devido ao uso do enxerto ósseo picado. Embora não tenha sido o objetivo do nosso estudo, pesquisas futuras devem incluir avaliação molecular para expandir nossa capacidade crítica e a produção científica de dados, bem como direcionar terapias farmacológicas potentes para induzir ainda mais a regeneração. A comparação entre enxerto ósseo autólogo local, enxerto ósseo autólogo distante e substitutos ósseos adjuvantes deve ser mais investigada nos processos de cicatrização óssea para alcançar o melhor tratamento possível para defeitos ósseos críticos.

## Conclusão

Este estudo evidenciou que diferentes técnicas para preparo e tratamento local de defeitos ósseos podem desempenhar um papel importante na indução da regeneração óssea. Utilizamos um modelo animal de defeito ósseo sem cicatrização espontânea durante todo o experimento. Por outro lado, nossos protocolos de enxerto ósseo autólogo local induziram regeneração óssea neste modelo animal. O enxerto ósseo picado gerou ossos recém-formados com maior massa, melhor microarquitetura e melhor integridade mecânica do que os ossos recém-formados após o enxerto ósseo macerado.
